# Smart Streets as a Cyber-Physical Social Platform: A Conceptual Framework

**DOI:** 10.3390/s23031399

**Published:** 2023-01-26

**Authors:** Theo Lynn, Charles Wood

**Affiliations:** 1 Irish Institute of Digital Business, Dublin City University, D09 Y5N0 Dublin, Ireland; 2 Collins College of Business, The University of Tulsa, Tulsa, OK 74104, USA

**Keywords:** streets, smart streets, sensors, cyber-physical systems, cyber-physical social systems, smart cities, platformisation

## Abstract

Streets perform a number of important functions and have a wide range of activities performed in them. There is a small but growing focus on streets as a more generalisable, atomised, and therefore more manageable unit of development and analysis than cities. Despite the public realm being one of the largest physical spaces on streets, the impact and potential of digitalisation projects on this realm is rarely considered. In this article, the smartness of a street is derived from the cyber-physical social infrastructure in the public realm, including data obtained from sensors, the interconnection between different services, technologies and social actors, intelligence derived from analysis of the data, and optimisation of operations within a street. This article conceptualises smart streets as basic units of urban space that leverage cyber-physical social infrastructure to provide and enable enhanced services to and between stakeholders, and through stakeholders’ use of the street, generate data to optimise its services, capabilities, and value to stakeholders. A proposed conceptual framework is used to identify and explore how streets can be augmented and create value through cyber-physical social infrastructure and digital enhancements. We conclude with a discussion of future avenues of research.

## 1. Introduction

By 2050, over 68% of the world’s population will live in urban areas [1]. As well as economic benefits, increased urbanisation presents significant challenges to governments and municipal authorities. Cities consume over two-thirds of the world’s energy and are responsible for over 60% of greenhouse gas emissions [1]. Furthermore, increased urbanisation can lead to significant urban health issues related to road traffic injuries, air and noise pollution, and barriers to safe physical activity, amongst others [1]. Against this backdrop, many urban areas are struggling with the strain urbanisation is putting on a decaying infrastructure [2]. In response, the concept of the smart city has emerged and gained traction over the last three decades; while there is an ongoing debate on the definition of a smart city, there is agreement that it involves the diffusion ofinformation and communication technology (ICT) to improve how different urban subsystems operate to meet the needs of people and communities [3,4]. The challenge with smart cities is one of scale. Working at city scale requires an often unprecedented investment of public funds, coordination, and a suitably long term horizon which presents significant governance, economic, and technology challenges, amongst others [5,6]. Furthermore, the focus on cities also neglects the needs of those who live in small and rural communities [7]. Unsurprisingly, streets have been proposed as a more generalisable, atomised, and therefore more manageable unit of development for improving urban subsystems and meeting the needs of both urban and rural communities [8,9].

Streets typically represent the largest portion of the public realm in towns and cities. As well as a thoroughfare for traveling from one point to another, streets play an important role in public health and safety, quality of life, environmental sustainability, social equity, and the economy [10,11,12]. Streets also play a less visible role; they incorporate much of the critical urban infrastructure to support towns and cities including, for example, telecommunication, water, energy, and waste [13]. More importantly, in the context of this paper, streets allow the live testing, experimentation, and evaluation of smart city technologies in a small-scale yet realistic setting.

The digitalisation of streets is an under-researched area and smart streets are at an early stage of maturity. This article stems from our reflection on the extant literature and the research challenges for smart city and CPSS projects, as well as our experience working on several digital town projects. This reflection suggests a dearth of conceptual tools to inform the envisioning of smart streets and related research projects, a prevalence of site-specific and use case-dependent conceptualisations and implementations that hinder wider generalisation, a lack of general design principles for integrating the social aspect in to intelligent public infrastructure, as well as a failure to consider CPSS from a multi-disciplinary perspective. The aim of this article is to raise awareness, stimulate discussion, and propose some initial avenues of research on smart streets. In this article, we make a number of contributions. Firstly, following a review of the smart street and CPSS literature, we extend the definition of smart streets to accommodate social networks between humans, computers, and humans and computers, and furthermore reflect the literature on CPSS. Secondly, we propose a novel general framework for conceptualising a smart street as a cyber-physical social platform and enabling the exploration of the complexity of a street as a system of systems without necessarily requiring adherence to a specific technological solution or reference architecture. In this way, it can be used to explore the concept of smart streets from multiple disciplinary perspectives. Thirdly, we scrutinise the literature on smart streets, digital platforms, and CPSS and elicit six avenues for future research on smart streets as cyber-physical social platforms.

The rest of this article is organised as follows. In Section 2, we discuss the evolution of streets to smart streets, the motivation for our conceptual framework. We then briefly discuss the nature of cyber-physical social systems and platforms in Section 3. In Section 4, we present our general framework for conceptualising smart streets as a cyber-physical social platform and explicate each of the components of a smart street. We identify and briefly discuss key implications of our framework as well as opportunities for future research including infrastructural and platform studies in Section 5 before concluding.

## 2. From Streets to Smart Streets

**Figure 1:**
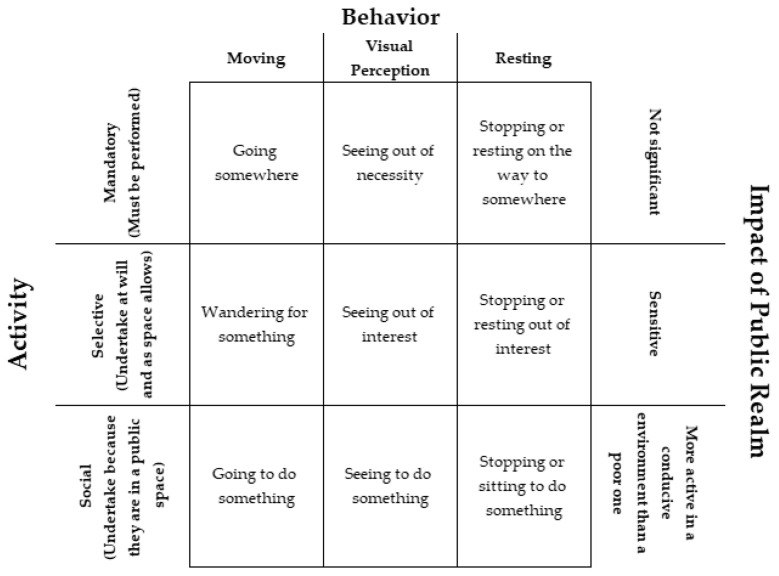
Categories of Human Behaviour in the Street (adapted from [14,16]).

As discussed above, streets are not merely thoroughfares that connect one point with another. As illustrated by Figure 1, the public perform a wide range of activities in streets that can be categorised as (i) mandatory (e.g., going to work or school and shopping), (ii) selective (wandering or sitting and watching street life), and (iii) social activities (having conversations) while human behaviour in streets can be classified as (i) moving, (ii) visual perception, and (iii) resting behaviours, which can occur discreetly, successively, or concurrently [14]. As such, it is a public realm that is actively and passively consumed depending on how it is structured as a public space. These structures highly influence the norms for how such a space is moved through and consumed by individuals or groups [15].

**Figure 2:**
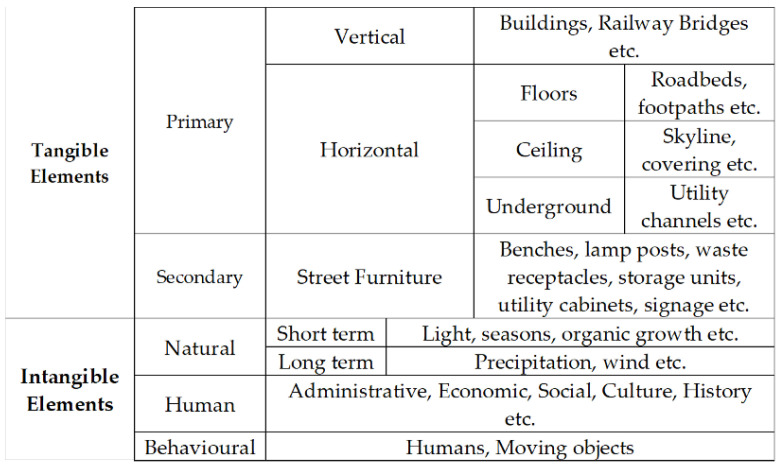
Tangible and Intangible Elements of a Street (adapted and extended from [16,20]).

Streets are multidimensional spaces from one property line to another and comprise a number of tangible and intangible elements that need to be taken into account (see Figure 2). Furthermore, they can be apportioned into three common zone types: the building edge, sidewalks, and roadbeds [11]. These zones may include distinct sub-zones and different design features and serve different functions. For example, sidewalks may include frontage (building edge), clear paths, street furniture, and buffers [11]. Sidewalks serve a transportation function in that they are both spaces of access, enabling people to move from one place to another facilitating access between properties and to people. They also serve a function for stationery activities, e.g., retail and infrastructure [17]. In addition to this, they play a critical bordering role providing citizens and pedestrians safety from vehicles and other risks [18]. Similarly, roadbeds may include transit facilities, ancillary lanes for cyclists or delivery vehicles, parking for motor vehicles and cyclists, and planting, amongst others [11]. Within these elements service street furniture and infrastructure are provided both on the surface and substrate. It is important to note that poorly planned streets can inhibit use and streets can be the site of conflict, anti-social behaviour, and undesirable activities [19].

Lynn et al. [9] define a smart street as

…a basic unit of urban space that leverages cyber-physical infrastructure to provide enhanced services to stakeholders, and through stakeholder use of the street, generates data to optimize its services, capabilities, and value to stakeholders.

Lynn et al. [9] proceed to define eight examples of smart street technology categories; namely, (i) connectivity, (ii) smart street information systems, (iii) traffic and transit management, (iv) accessibility, safety, and security, (v) smart street furniture, (vi) climate protection, environmental monitoring, and weather mitigation, (vii) environmental sustainability, and (viii) other technologies that encourage street activity [11]. Table 1 defines each category and provides examples with reference to the extant literature. It is important to note that these technology categories are not mutually exclusive and may complement or even depend on each other.

While it is inferred from this definition and the associated technology categories that the street create value through stakeholder engagement, the definition is ambiguous with respect to two inter-related issues: (i) social interaction and (ii) the degree to which the street is an open or closed loop system. Firstly, we argue that given the range of human behaviours and activities on a street, the social interaction between different human actors, between human actors and technical artifacts, and between computers as social actors needs to be more explicit. Secondly, Cassandras [21] has argued that to (i) avoid unintended consequences (and presumably malfeasance), (ii) provide intelligent support for decision making, and (iii) integrate humans in the loop while recognising human actors may have different, potentially conflicting, motivations requires governance and therefore a closed loop. Accordingly, Cassandras [21] recommends that municipal governments view smart city systems as cyber-physical social systems (CPSS) when developing and implementing the policies necessary to provide incentives and deliver the value of CPSS to smart cities.

**Table 1:** Smart Street Technology Categories (adapted and extended from Lynn et al. [9]).

Category	Description	Sample Smart Street Technologies
Connectivity	The provision of a substrate of network connectivity, power, and associated hardware ideally underground or integrated seamlessly into other street objects.	- 5G and 6G network infrastructure to support intelligent vehicle mobility and smart street applications [22,23,24,25,26] - Community Wi-Fi, municipal wireless mesh networks, or blockchain-based peer-to-peer wireless network to support free public Wi-Fi [27,28]. - Reconfigurability of public space, e.g, automated retractable power units [29] - Smart street furniture with built in Wi-Fi, telecommunications, and switchboards [30,31].
Smart street information systems	Information systems measuring, analysing, modelling, and visualising data generated on and by smart streets to support and actuate decision making.	- Urban data platforms incl. open data management systems [32] - ICT as a planning support [32] - Strategic urban planning [32] - Traffic control systems [32] - Traffic demand management [32] - Energy demand response [32] - Mobile applications for citizens [32] - Neighbourhood energy management systems [32]
Traffic and transit management	Management and optimisation of multi-functional street use including dynamic user prioritisation and street use change [9,29].	- Automated street bollards, license plate recognition, and embedded road lighting to prioritise users and manage transportation, change street use, and record infringements [9,29,33]. - On-street parking sensors for identifying vacant spots, charging, recording usage, and signalling pricing [34]. - Autonomous vehicles to support freight and micro-mobility, e.g., delivery systems [35,36].
Accessibility, safety, and security	Use of technology to identify and eliminate obstacles and hazards, provide multimodal signals to alert those in need, contact emergency services, and otherwise deter unwanted behaviour or identify unwanted activities [37,38,39].	- Bluetooth beacons that provide audio or text messages to smartphones or local visual signals to alert those in need [37]. - Object detection systems to identify unpermitted obstructions, potholes, water pooling, or other seasonal or anomalous issues without first notification from the public [40,41]. - Micro-mobile autonomous vehicles for transport over short distances [36]. - Security cameras systems supported by machine learning to monitor speeding vehicles, prevent crime, support access management, and enable payment transactions [38,42]. - Emergency service communication capabilities integrated into smart furniture [39].
Smart street furniture	Multi-functional street furniture designed as an active part of the street experience supporting different activities and behaviours to meet desired outcomes.	- Smart lampposts with LED smart lights and built-in GPS, Wi-Fi, telecommunications and switchboards, CCTV, telemetry, EV charging points, and NEMA controllers for traffic signals and pedestrian crossing [30,43,44]. - Smart kiosks that can serve as multifunctional points for sharing information, completing transactions and payments, communicating with emergency services or other third parties, relaying or providing access to the Internet, device charging, research collectors, and advertising [39,45] - Hybrid and solar-powered smart benches with integrated shelter and lighting, CCTV, USB and EV charging, bicycle parking and services, and video displays for information, advertising, and entertainment [39,46,47]. - Hybrid and solar-powered waste solutions including autonomous robots and waste collection systems with sensors to signal the need for collection [39,48]. - Electronic storage units for extending collection and delivery beyond normal working hours [49]. - Other smart furniture including public toilets with smart access management and intelligent wash disinfection and smart public drinking fountains [50].
Climate protection and weather mitigation	Sensor-based systems that monitor decay in physical materials or actuate weather mitigation strategies that (i) block wind, (ii) provide shelter, or (iii) provide shade [51].	- Sensor-based systems for monitoring street infrastructure decay [52]. - Sensor-based retractable sidewalk awnings [51].
Environmental sustainability	Information systems and technologies that measure, analyse, model, and visualise data generated on energy harvesting systems and the evolution of the environment in a street smart street, and support and actuate decision-making that supports environmental policies [32].	- Sensor-based environmental monitoring and prediction systems [44]. - Programmable, flexible, and adaptive systems for prioritising street use, e.g., transit and parking [29]. - Energy harvesting technologies integrated into street furniture, roads, and sidewalk pavements, and railways to power street utilities [53,54,55,56].
Other technologies that encourage street activity	Information systems and technologies that invite street activity, increase desirable street behaviours and activity, or encourage new street behaviour and activity.	- Interactive smart glass that converts storefront window displays into multimedia displays [57,58]. - Geo-fencing and street furniture integration to increase sense of enclosure in streets and communicate with street users [59]. - User of conversational technologies, integrated with SMS and QR codes, to transform passive street furniture into a social experience [60,61,62,63].

## 3. Cyber-Physical Social Systems and Platforms

Our conceptualisation of smart streets brings together concepts from two emergent literature bases; namely, cyber-physical social systems and platforms, into a general conceptual framework.

### 3.1. Cyber-Physical Social Systems

In the last three decades, we have seen the emergence of the Internet of Things (IoT) and with it a renewed and increased interest in cyber-physical systems (CPS). Such systems integrate computation with physical objects and processes, a literal co-mingling of the physical world and the cyber world (including computation, communication, and control systems) [64]. CPS has been cited as the computation substrate that will connect future public critical infrastructure to intelligent systems and software [7]. More recently, the literature on CPS has expanded to integrate social systems, bridging the gap between human intelligence and machine intelligence by including a social domain characterised by human participation and interactions [65]. In such cyber-physical social systems (CPSS), humans, software, and physical objects (through sensors) are linked through a CPSS to meet a given actor’s social interaction demands and react to the physical world [65]. Central to the concept of CPSS is at least one physical component responsible for sensing and actuation, one cyber component for computations, and one social component for actuating social functions [66]. Place is an important and increasingly complex construct in the CPSS literature, including physical spaces, virtual spaces, social networks [65,67], and the overlay of these spaces through technologies such as augmented and extended reality. Given the role of purpose and place in CPSS, context awareness is a critical component of CPSS [65]. Commonly cited CPSS use cases are unsurprisingly related to places, including smart homes, but also to larger urban spaces, e.g., smart cities [21,65,68,69]. Indeed the latter has attracted the attention of leading technology companies worldwide, most notably and somewhat controversially, Google’s Sidewalk Toronto project [51].

### 3.2. From Product Platforms to Digital Platforms

Platforms and the related term, platformisation, are widely referenced in both the scholarly literature and the media, while once platforms were largely defined from a production or computational perspective, they increasingly have wider political, figurative, and architectural connotations [70]. Meyer and Lehnard [71] define product platforms as

…a set of subsystems and interfaces that form a common structure from which a stream of derivative products can be efficiently developed and produced.

In this conceptualisation, a product architecture is the combination of subsystems and interfaces [71]. What distinguishes a platform architecture from a product architecture is its capacity to enable the creation of derivative products [71], while Meyer and Lehnard note that services, both in the real world and online, are not inconsistent with this conceptualisation of the platform or platform architectures [71], their conceptualisation infers a finished product or completed service. More recently, we have seen the emergence and adoption of Web 2.0 and the so-called Third IT Platform, while the former emphasised the role of users through co-creation, participation, ease of use, and interoperability [72], the latter heralded a cyber-physical future that emphasised interdependencies between mobile computing, social media, cloud computing, information/analytics, and the IoT [73]. Here, as Ramaswamy and Oczan [74] note, digitalised platforms differ in that:

…the offering is no longer “finished” in the traditional sense, and the creation of value continues in a joint space of interactional value creation, between engaging actors (often consumers and their social networks) interacting with organizing actors (often the firm and its associated organisational ecosystem). The traditional notion of offerings as goods and services to be optimized in terms of a fixed set of features and attributes is inadequate in connecting with the new opportunities for creating value in an age of digitalized interactions.

This wider conceptualisation of a platform is one in which a multitude of actors can interact with digital systems and one another to create value. In this way, the platform is a multi-sided network in which goods, services, and increasingly data are exchanged between the actors to create value [75]. In addition to providing an enabling infrastructure and system core, the platform plays a vital role mediating between different groups of actors [75,76]. While platforms can be merely conceived as product platforms in line with Meyer and Lehnard [71] in that they provide an extensible codebase to which third party modules can be added [76], the socio-technical view of digitalised platforms conceives the platform as comprising technical elements (software and hardware) and associated organisational processes and standards [76]. The agency of the user is a critical difference between non-digital and digital platforms. As de Reuver et al. [76] note, non-digital platforms assume a stable core and a variable periphery governed by an overall design hierarchy typically determined by the platform owner or sponsor, but digital platforms are not necessarily constrained by such design hierarchies. The separation of concerns combined with the ability to reprogram, re-edit, and re-use data and code, particularly in the context of open source software and open data, enables platforms to evolve and new applications to emerge in ways often unplanned and unexpected. Indeed, the generative dynamics of digital platforms, particularly when coupled with openness, are seen not only as a key enabler of the platform evolution but as a critical success factor in adoption [77].

Poniatowski et al. [75], building on de Reuver et al. [76] and Van Alstyne et al. [78], conceptualise digitalised platforms as comprising three layers—platform infrastructure, platform core, and platform periphery. Infrastructure implies an underlying socio-technical system characterised by ubiquity, reliability, invisibility, gateways, and breakdown [79]. Similar to other infrastructures, for example electricity grids, it is defined by control. Similarly, platform infrastructure is the foundation of any platform, is largely hidden from third parties, and is controlled by the platform sponsor [75]. The platform core sits on the platform infrastructure and is controlled by the platform core owner, who may or may not be the platform owner [75]. Third parties participate and contribute to the platform through the platform periphery, again controlled by the platform owner [75]. This model can be illustrated by reference to Amazon. Amazon both are the platform sponsor for Amazon Web Services and the platform core that comprises Amazon.com, which includes Amazon’s own retail business but also a periphery comprising other retailers and service providers. It is important that a platform may have multiple platform cores. Again, in the context of Amazon, Amazon Web Services leverages Amazon platform infrastructure to support its cloud business which comprises platform-as-a-service, software-as-a-service, etc. This infrastructure is both used by Amazon and by a wide range of third parties. Table 2 summarises the key concepts of digital platforms.

## 4. A Conceptual Framework of Smart Streets as a Cyber-Physical Social Platform

**Figure 3:**
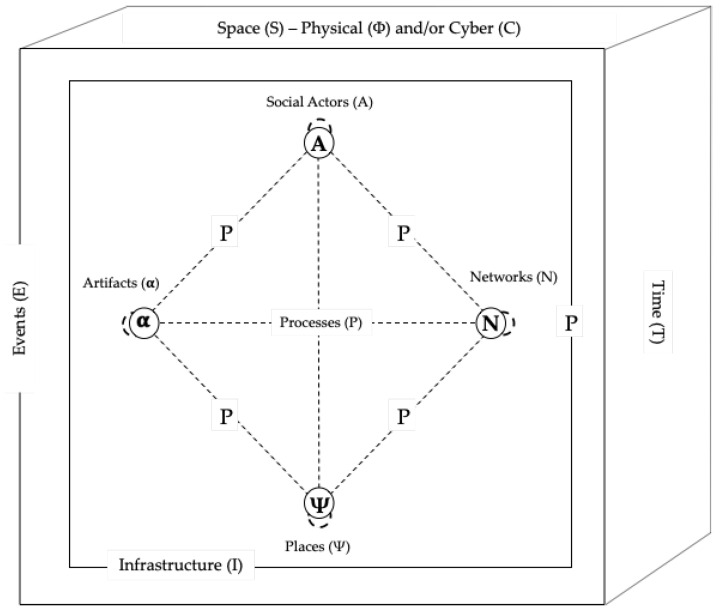
A conceptual framework of smart streets as a cyber-physical social platform.

Unlike purely digitised platforms, the term ‘cyber-physical social platform’ implies a platform infrastructure comprising physical and cyber platform elements upon which a platform core resides, that can enact physical, computational, and social processes by itself or through the interaction of other entities through the platform periphery. Figure 3 presents a general framework for conceptualising smart streets as a cyber-physical social platform. This conceptual framework is general in that it is capable of being used to understand and explore smart street-related research questions or problems in conjunction with widely accepted levels of generalisation (abstraction) in different academic disciplines, including both the social sciences and computer sciences. Addressing the issues with earlier definitions of smart streets [9], we assume an updated definition of smart streets that accommodates social networks between humans, computers, and humans and computers, and reflects the literature on CPSS. However, while a closed loop is most likely desirable from the perspective of municipal authorities who have a legal responsibility for the public realm that is the street, it leaves the issue of whether the system per se is open or closed, undetermined in order to support a general level of abstraction for theoretical and practical exploration. Accordingly, we define smart streets as a basic unit of urban space that leverages cyber-physical social infrastructure to provide and enable enhanced services to and between stakeholders, and through stakeholder use of the street, generates data to optimise its services, capabilities, and value to stakeholders. The proposed conceptual framework provides a sufficiently general abstraction of smart streets to facilitate sense making without getting into a non-generalisable level of granularity or worrying about specific definitions of smart streets or indeed cyber-physical social platforms.

In this framework, five core entities are identified and defined: Social Actors, Artifacts, Networks, Places, and Infrastructure:

- **Social Actors (A)** are any agents who possess (i) a common cognitive reference frame and (ii) the specific competence for understanding, accepting, and dealing with the common cognitive reference frame, the actor itself, and other entities [87]. These may include individuals or groups of humans, as well as the computer as a social actor. It is important to note that individuals can play different and multiple roles with respect to a given street, e.g., as residents, owners, consumers, travellers, etc. Similarly, groups of individuals may be in organisations or movements with different degrees of connectivity and formality. Given that the public realm is typically the responsibility of a municipal authority or other governmental agency, they are most likely both the platform sponsor and platform owner but this need not be the case. For example, there are numerous examples of private streets and roads in developments (e.g., university campuses, large private retail villages, housing developments, etc.) that may be controlled or managed by a private entity.
- **Artifacts** (α) are objects that enable interactional creation of value by the agency by engaging a social actor who constructs outcomes of value in different contexts giving rise to experiences that may be subjective to each person or objective depending on the nature of the Social Actor (adapted and extended from Ramaswamy and Ozcan [74]). These include any physical or virtual object, e.g., wearables, information, street furniture, roadbed, sensors, vehicles, utility infrastructure, computer hardware, etc.
- **Networks (N)** are systems of interconnected entities and are both conduits and entities in themselves. These may be social networks in the traditional sense (e.g., networks between people) or communications networks, including sensor networks. It is important to remember that streets play an important role linking adjoining networks including streets and buildings but also utilities.
- **Places (ψ)** are psychologically meaningful domains where identifications of Social Actors to locations are formed through the sharing of experiences within a space and socially co-constructed through repeated interactions [88]. In this respect, they are distinct from a location in space-time. Places (ψ) may be located in physical space (Sϑ) and cyberspace (SC). In the context of streets, it is important to note that while much attention is placed on the physical surface of a street, many streets are multi-level and also contain substrates, which can generate, capture, and consume data.
- **Infrastructure (I)** is the basic cyber-physical and organisational structures, systems, and facilities that support the sustainable functionality of the street. Infrastructure may collect data and metadata that are byproducts of indirect and/or passive street use. For example, the street may be considered part of the cyber-physical infrastructure if there are sensors capturing data about road use.

Each of these five core entities may be physical or cyber in nature. It is important to note that as each of these entities can, although not necessarily, in themselves be a system, a smart street is in effect a system of systems. Ramaswamy and Ozcan [74] treat an interface as a discrete entity and define it as:

…a point of connection between hardware, software, data, and individuals, whose representations and manipulations in relation to each other produce the possibility of interaction, providing multiple modes and means of communication and translation between the external and the internal.

Unlike Ramaswamy and Ozcan [74], we treat interfaces as being an essential property of an entity as per the theory of systems, as opposed to a discrete entity. In this way, the general abstraction is maintained.

In streets, entities can be affected by processes and events, both of which have defined starts and finishes:

- **Processes (P)** are a series of actions, motions, or operations leading to a change in the state of an entity; they are changeable, dynamic, and have a start and finish [89]. Processes determine how entities interoperate and may comprise general and domain-specific processes. As illustrated in Figure 1 and Table 1, a wide range of behaviours and activities take place on streets, all of which are capable of some form of data capture, digital optimisation, or transformation. Processes play a key role in facilitating the interactional relations between different entities which take place in infrastructure situated in space and time.
- **Events (E)** are occurrences of interest at a given time in space, physical and/or virtual, with a specific start and end time. A wide range of events, of different scales, occur on (e.g., protests, festivals, parades, and fairs) and impact streets (e.g., weather events or public health restrictions).

Entities exist and processes and events occur in space and time:

- **Space (S)** in this context includes physical space and cyberspace. Physical space is the unlimited expanse of the universe [90], in which all material objects are located and all phenomena occur [91]. Strate [92] conceptualises cyberspace as events involving relationships between humans and computers, between humans through computers, and between computers themselves. It should be noted that these definitions are sufficiently abstract to accommodate a wide range of combinatorial and discrete cyberspace conceptualisations. Of significant relevance in the context of the smart street CPSS is that of perceptual space, a building block of cyberspace, which Strate [92] defines as the the sense of space generated by the computer–user interface, through one or a combination of our senses. Such perceptual spaces sit between and bridge physical and cyber space, and includes augmented reality and hyper-reality.
- **Time (T)** is a point in time in the indefinite continued progress of existence and events in the past, present, and future, regarded as a whole, as measured by Coordinated Universal Time [93].

It is important to note that while spaces may exist in physical and/or cyberspace, the passage of time in both has a firm basis in objective reality [92].

## 5. Towards a Future Research Agenda on Smart Streets as Cyber-Physical Platforms

The early stage of conceptualisation of smart streets as cyber-physical social platforms presents a cornucopia of research across multiple disciplines, from computer sciences to urban engineering, cognitive sciences, social sciences, and business disciplines. In this section, we discuss six areas that we call for further research on; namely, conceptual ambiguity, design principles, technological challenges, sustainability and value generation, trustworthy smart streets, and methodological issues.

### 5.1. Conceptual Ambiguity

The concept of smart streets as cyber-physical social platforms presents a number of conceptual challenges. Firstly, while borrowing from the smart city literature, the concept of a smart street is relatively new and evolving. Further work is required to frame the boundaries of a smart street and develop taxonomies and typologies of streets and street zones and the applicability of different smart street solutions to these different types and zones. For example, the uses and needs of streets in residential and industrial areas are different, as are those for highways, main streets, and service lanes.

While more evolved, CPSS is still an emerging concept. In their review of the literature, Yilma et al. [66] note some salient issues with the conceptualisation of CPSS. These include inconsistent definitions of CPSS, use case-dependent conceptualisations of CPSS leading to generalisation issues, and a lack of design principles that integrate the social aspect to the underlying core CPS. These issues are likely to be further exacerbated in a nascent use case such as smart streets.

With respect to digital platforms, two major theoretical approaches have emerged: infrastructural studies and platform studies [79]. Plantin et al. [79] note that, while both infrastructure and platform refer to structures that underlie or support something more salient, infrastructure studies have focussed on widely shared socio-technical systems characterised by ubiquity, reliability, invisibility, gateways, and breakdown, while (digital) platform studies have focused on how hardware and the software environments affect the characteristics of the application software built upon them. As a result, the latter is more concerned with programmability, affordances and constraints, connection to heterogeneous actors, and accessibility of data and logic through application programming interfaces [79], while this demarcation was once clear, Plantin et al. [79] note that there is some ambiguity of the relationship between them, particularly with the advent of hyperscale cloud computing; many digital platforms provide widely accessible services of public value. Smart street platforms reflect this duality; they are part of the underlying infrastructure of the public realm and should have the characteristics of such a public utility, and yet much of the value of a smart street is derived from the platform attributes. Mynatt et al. [7] note that two significant challenges for intelligent public infrastructure are that (i) cities, communities, and municipalities lack the expertise and financial resources of industry to progress the technologies and applications necessary for intelligent infrastructure and (ii) integration of intelligent infrastructure into incumbent systems while mitigating interruptions, reducing exposure to threats, and ensuring continuity of service is problematic. The characteristics and benefits of a platform approach that relies on programmability, accessibility, and extensibility may be inconsistent with the reliability and security inherent in critical infrastructure.

### 5.2. Design Principles

Further research is required with respect to the design principles necessary to inform the design of a smart street CPSS platform architecture. At a high level, Mynatt et al. [7] suggest that intelligent infrastructure draws from basic research and advancements in (i) CPS, (ii) artificial intelligence (AI), machine learning, and data analytics, (iii) security, safety, and privacy, (iv) networking, (v) systems programming, (vi) decision support, and (vii) citizen support. They further stress the need for interoperability between intelligent infrastructure, legacy systems, and third parties [7]. From a different although not inconsistent perspective, conceptualised as a CPSS platform, a smart street can be viewed as a system of systems (SOS) or complex adaptive system [66,94,95]. The insights of Maier [95] are informative, suggesting four architecting principles for SOS; namely, stable intermediary forms, policy triage, leverage at the interfaces, and ensuring cooperation. From an SOS perspective, Yilma et al. [66] have proposed that aligning a CPSS, in this case a smart street platform, with the theory of systems assists the design process by defining the systemic properties of each interacting entity, e.g., relations, behaviours, functions, structures, objectives, interfaces, environments, and system components. Existing CPSS and IoT reference architectures may be informative (see, for example, [9,96]); however, care needs to be taken that the system is not designed in isolation.

### 5.3. Technological Challenges

There are a wide range of technological challenges inherent in a smart street CPSS platform, far too many to be addressed within the constraints of this article. Notwithstanding this, we call out three specific technological challenges. Firstly, people are essential to the success of a street and a CPSS. In both contexts, humans are both service providers and service consumers, and can interact as individuals and groups. For example, Zhou et al. [96] note that they both play a role as individuals in citizen sensing and citizen actuation, and similar in formal and informal groups through crowdsourcing and crowdsensing. In the context of streets, citizens can provide data through their technology artifacts (e.g., smartphones, vehicles, or other sensors) and can either use their own or public actuators (also artifacts, e.g., smart pavements) to enact processes and affect the environment around them. This may be both active and passive. However, humans are not the only social actors; the computer may also be a social actor. This presents new opportunities for socialisation research. For example, Yilma et al. [66] introduce the concept of socially capable CPSS devices; socialised machines that learn from and interact with humans. To deliver on the potential of such devices requires research on not only the learning process that enables a machine to detect and reason social interaction responses but also empowering machines to respond in a desirable manner through social actuation [66]. Furthermore, one can imagine different types of relations between humans, machines, and the smart street system per se. In the design of a smart street CPSS platform, the behaviour description model needs not only to understand the role, function, and behaviour of human actors [96] but also all potential social actors on a street from a system perspective. As Figure 1 and Yilma et al. [66] imply, this is made more difficult when one considers personalisation and the need for a smart street CPSS and/or socialising machines to respond to the personal needs of a specific social actor. While personalisation, in general, is a long-established field in computer science, personalisation in CPSS and street environments is largely unaddressed.

Secondly, as can be seen in Table 1, there are a wide range of technologies, use cases, or applications for smart streets. Much of the existing CPSS literature addresses use cases relevant to smart streets, including transportation, energy management, environment and sustainability, tourism, and hospitality [96]; however, while there has been considerable research undertaken on smart city- and street-related technologies, these are largely designed as discrete elements of a system, and the interoperability of these systems, from a pure research and real world perspective has not been considered comprehensively. To this end, there is a need for a living lab, a smart street lab imbued with smart street technologies and real people, where a smart street CPSS platform can be experimented with, tested, evaluated, and optimised.

Thirdly, and relatedly, the enabling infrastructure and technologies to support a smart street CPSS platform are considerable. They include computing infrastructure (cloud, fog, mist, and edge computing), communications and (sensor) network technologies, sensors and actuators, computational techniques (including machine learning and deep learning), as well as other related enabling applications (e.g., social network sites) [96]. The nature of streets and the range of potential social actors that might engage with a smart street platform infers a highly dynamic, uncertain, and heterogeneous environment. To meet service levels, a smart street CPSS needs to be integrated, coordinated, and optimised from the cloud to the edge, which alone may prove a fruitful avenue of research [97]. Furthermore, smart cities are a much cited use case for next generation wireless systems, for example, 6G [22,23,24,25,26]. As such, future research should consider the benefits and challenges of such infrastructure and specifically the use of novel techniques for the deployment and optimisation of resources to ensure quality of service requirements for smart streets [24,98,99].

### 5.4. Sustainability and Value Generation

Research suggests that smart street infrastructure projects are not possible without public financing (see, for example, [100]). Similarly, Mynatt et al. [7] note that sustainability is a formidable barrier to the long-term success of intelligent infrastructure projects such as smart streets. This is partly due to a dearth of novel economic models that recognise the value of certain interventions and differences in upgrade cadence between traditional infrastructure (upgraded over decades) and ICT (upgraded over years). With respect to the latter, Mynatt et al. [7] suggest that some interventions that result in desirable outcomes are abstract and do not generate income or reduce costs, e.g., cleaner air or reduced crime. How we evaluate smart street CPSS platforms therefore requires an understanding of how social actors and platform sponsors come together to co-create value that would not otherwise materialise without additional public sector investment, a concept referred to as additionality [101,102,103].

In smart streets, a wide range of stakeholders who interact with a street can implement and benefit, from not only the smart street platform, but from a wider ecosystem of technological artifacts, including other digitalised interactive platforms (DIPs) to co-create value for themselves and other stakeholders. Ramaswamy and Ozcan [74] argue that DIP’s afford new ways in which value can be generated through exchange or usage of resources and processes in activities but through interactions, creational and otherwise. As the range and volume of interactions increase between entities, there is greater potential value creation for stakeholders but also for evolving and optimising the platform. For example, typically the public realm in a street is managed by a local authority and therefore a central consideration in the adoption of smart street technologies and an underlying platform is that it will positively affect everyday life on the street and support desirable national and local policy outcomes. As can be seen from Table 3, smart street technologies can contribute to different outcomes, including economic, environment, human health and safety, social inclusion, and scientific and education policies. Smart street technologies generate value from the use of data and through data. The more technologies that are available to stakeholders for use, the more potential data is generated, providing the opportunity for greater overall value. Empirical research is required to explore the various types of interactional relations that a smart street enables and that occur and how value is created and for whom.

### 5.5. Trustworthy Smart Streets

In a non-digital sense, people do not typically need to consider whether they trust the “street”. In conceptualising the smart street as a CPSS, consideration of how to build trust in such systems and how to respond when trust is violated is critical. Intelligent infrastructures including smart streets are enabled by data collection, processing, transfer, and use, while these offer communities a myriad of potential beneficial outcomes, they raise significant concerns regarding data confidentiality, integrity, accessibility, and government surveillance [104]. These fears are not unfounded. Recent research found that in the context of smart street furniture, through omission or lack of clarity, silences surrounded how data generated was captured, aggregated, shared, and used [31].

Trust is accepted as one of the major barriers to technology adoption. It is generally defined as a willingness to accept vulnerability based on positive expectations of another party [105]. It infers a psychological state of willingness to be vulnerable, representing a volitional choice or decision and a positive expectations of another party [106]. Trustworthiness is typically understood as the perception of another party along three sub-dimensions: (i) ability, (ii) integrity, and (iii) benevolence [107]. These sub-dimensions have been applied in an ICT context as (i) accuracy, capability, and functionality, (ii) reliability and consistency of performance, and (iii) helpfulness and responsiveness [108,109,110]. Unsurprisingly, there is a dearth of research on trust in CPSS, smart streets, and associated technologies. The conceptualisation of smart streets as a CPSS raises some interesting questions for trust researchers. Firstly, (smart) streets are consumed by active and passive users. As such, they may not be aware of the *smartness* of the street and yet their data may be captured. Echoing Lopresti and Shekhar [111], who is the user to trust? The street? The local authority? The vendor who installed the system? How should the *smartness* of the street be communicated? Is it possible to opt out from latent data capture on the street? How should/can data privacy and protection in a smart street be regulated? Recent research on control frameworks for assurance and accountability may be informative [112]. Secondly, and as noted in Section 5.3, smart streets assume interactions between a wide range of social actors including human individuals and groups, organisations including industry and government, and non-intelligent and intelligent machines. As such, a trust model for smart streets as a CPSS will need to integrate concepts from the literature on interpersonal, organisational, and technological trust. Thirdly, our conceptualisation trust in artificial intelligence is still at an early stage. If, as discussed, we anticipate that socialised machines will learn from and interact with humans and other machines, we need to consider how such machines can learn what it means to trust and be trustworthy. While formalisms are widely used in a wide range of disciplines, the interdisciplinary application of formalisms in the context of trust and AI is limited. Those formalisms that do exist, typically in computer science, do not take in to consideration advances in the psychology and information systems literature. Again, for trust, CPSS, and smart street researchers, this may provide a fruitful avenue of research.

The issue of security is highly correlated and often conflated, rightly or wrongly, with trust in computing. As discussed, smart streets not only assume computing operations from the cloud to the edge but incorporate intelligence in edge computing systems. This significantly increases the potential attack surface from a security perspective, opening up smart street systems and associated actors to a wide range of direct and indirect attacks including distributed denial of service (DDOS) attacks, side channel attacks, malware injection attacks, and authentication and authorisation attacks [113,114]. Edge AI, in particular, is used for inferencing based on pre-trained models, evasion attacks using adversarial samples, and privacy attacks to siphon off valuable information from the data used by a particular AI model [114]. There is a significant research opportunity in mapping potential smart street security threats and developing appropriate hardened countermeasures.

### 5.6. Methodological Issues

Smart street platforms will encounter similar methodological issues to all digital platforms. Our conceptualisation of smart streets as CPSS platforms infers a level of complexity, dynamism, interaction between entities, and information volumes where traditional approaches to computer science and information systems research built on reductionism may not be appropriate [115,116]. At a purely technical level, platforms, in general, and specifically in a smart street context, are built upon, interact with, and integrate with other platforms and technologies. As de Reuver et al. [76] note, this presents both vertical and horizontal scoping challenges, as well as more general methodological challenges. Approaching smart street platforms at only one level or layer of an architecture risks misunderstanding how different design decisions affect one another while focussing on one specific application, as is often the case in extent smart city technology research and risks misunderstanding how different entities in a smart street affect each other and outcomes [76]. Similarly, similar to general digital platform research, as well as challenges presented by complexity, smart street research may face methodology challenges including difficulties isolating units of analysis, insufficient study time horizons or short-termism, and bias towards successful, popular, or prominent applications or case sites [76]. These challenges can result in a lack of comparability between studies and inadequate understanding not only of causalities but of how the wider system interoperates [76]. Indeed, a contribution of our conceptual framework is to help researchers visualise smart streets in such a way as to overcome some of these challenges and structure research in a systematic way ideally in one single smart street platform.

## 6. Conclusions

The diffusion of ICT to improve the subsystems in the lived environment and meet the needs of people and communities is only going to increase in importance and proliferation. Research on so-called smart city technologies and cyber-physical social systems is hindered by reductionist approaches and access to real-world city-scale testbeds. In this article, we focus on the street as a more feasible starting point and building block for smart city research. We make three primary contributions. Firstly, following a review of the smart street and CPSS literature, we extend the definition of smart streets to accommodate social networks between humans, computers, and humans and computers, as well as reflecting the literature on cyber-physical social systems. Secondly, we propose a novel general framework for conceptualising a smart street as a cyber-physical social platform that integrates concepts from smart streets, digital platforms, and the cyber-physical social system literature. Thirdly, we elicit and discuss six avenues for future research on smart streets as cyber-physical social platforms that addresses gaps and failings in existing computer science, social science, and IS research. The underlying motivation for this article has been to raise awareness, stimulate discussion, and propose some initial avenues of research. In this respect, we believe the concept of smart streets as cyber-physical social platforms opens up exciting new avenues for research, not only for computer scientists, but those from urban engineering, cognitive sciences, and social sciences to collaborate in an inter- and multi-disciplinary way to explore and populate with clarity and depth. 

## Figures and Tables

**Table 2:** Key concepts on digital platforms.

Term	Definition
Digital platform	An extensible codebase to which complementary third-party modules can be added (technical view) or technical elements (of software and hardware) and associated organisational processes and standards (socio-technical view) [76].
Generativity	The capacity to produce unanticipated change through unfiltered contributions from broad and varied audiences [80].
Multi-sided platform	A business model that enables interactions between two or more distinct sides where each side is affiliated with the platform [81]. Typically, multi-sided platforms are characterised by network externalities [82].
Multi-sided market	Two or more groups or users interact through an intermediary or platform and where the decisions of each group or user affects the outcomes of another group or use, typically through an externality, and platforms are price setters on both sides of the market [82,83,84].
Network externality	The utility that a given user derives from a good depends upon the number of other users who are in the same network [85], while the value of a direct network externality depends on the number of users within the same user group and the value of an indirect network externality depends on the number of users in a different user group, i.e., the net utility on side “*i*” increases with the number of members on side “*j*” [76,86].
Platform infrastructure	A level of platform abstraction that forms the foundation for the platform core. In digital platforms, it is the underlying socio-technical systems controlled by the platform sponsor and upon which the platform core sits [75].
Platform core	A level of platform abstraction that sits on the platform infrastructure and is controlled by the platform core owner. Third parties can interact with the platform core [75].
Platform periphery	A level of platform abstraction that represents the contributions to the platform core provided by third parties (complementors) that typically complement the platform core and may form an ecoysystem [75].

**Table 3:** Selected policy outcomes mapped to smart street technology and policy categories.

Selected Policy Outcomes	Smart Street Technology *	Health and Human Safety	Quality of Life	Economic	Environment	Social Inclusion
Increase in connectivity	CO					
Reduction in energy, CO2, GHG, and other emissions	TTM, SSIS, SSF, GI, OSA					
Reduction in fossil fuel dependence and energy security	TTM, SSIS, SSF, GI, OSA					
Reduction in air, noise, and light pollution	TTM, SSI, SSF, GI, OSA					
Increase in quality and quantity of environmental data	SSIS, ACC, GI					
Increase in awareness of green issues	TTM, SSF, GI					
Reduction in traffic related congestion and emissions	TTM, GI, ACC					
Increase in public realm flexibility, use, and intensity	CO, SSIS, TTM, SSF, GI, OSA, ACC					
Increase in destination attractiveness and visitor traffic	CO, SSIS, TTM, ACC, SF, OSA					
Increase in incumbent commercial activity	CO, SSF, GI, OSA					
Stimulation of new social and commercial businesses	CO, SSIS, TTM, SSF, GI, OSA					
Increase in employment	CO, SSIS, TTM, SSF, GI, OSA					
Increased property security	SSIS, ACC					
Increase in property value and rents	CO, SSIS, GI, OSA, ACC					
Reduction in traffic related injuries and health related costs	SSIS, TTM					
Improved urban planning and management	SSIS					
Increased road safety and optimised parking	SSIS, TTM					
Increased personal safety	SSIS, TTM, ACC					
Increased access to physical activity	CO, TTM, ACC, GI					
Increased availability to high quality open data	CO, SSIS, TTM, SSF, GI, OSA, ACC					

* Key: CO: Connectivity; SSIS: Smart Street Information Systems; TTM: Traffic and Transit Management; ACC: Accessibility, Safety, and Security; SSF: Smart Street Furniture; GI (Green Infrastructure): Climate Protection and Weather Mitigation and Environmental Sustainability; OSA (Other Street Activity).

## Data Availability

Not applicable.
